# Correlates of self-report chronic insomnia disorders with 1–6 month and 6-month durations in home-dwelling urban older adults - the Shih-Pai Sleep Study in Taiwan: a cross-sectional community study

**DOI:** 10.1186/s12877-016-0290-6

**Published:** 2016-06-03

**Authors:** Jing-Hui Chiou, Hsi-Chung Chen, Kuang-Hung Chen, Pesus Chou

**Affiliations:** Department of Family Medicine & Center for Geriatrics and Gerontology, Taipei Veterans General Hospital, Shipai Rd., Beitou Dist. Taipei City, 112 Taiwan; Department of Psychiatry & Center of Sleep Disorders, National Taiwan University Hospital, No. 1, Chang-Te St., Zhongzheng Dist. Taipei City, 100 Taiwan; Center of Neuropsychiatric Research, National Health Research Institutes, 35 Keyan Road, Zhunan, Miaoli County 350 Taiwan; Community Medicine Research Center, National Yang-Ming University, Linong St., Beitou Dist. Taipei City, 112 Taiwan

**Keywords:** Chronicity, Insomnia disorder, Older adults, Shih-Pai Sleep Study

## Abstract

**Background:**

To examine the correlates of insomnia disorder with different durations in home-dwelling older adults.

**Methods:**

A cross-sectional survey in the Shih-Pai area of Taipei City, Taiwan (The Shih-Pai Sleep Study). A total 4047 subjects over the age of 65 years completed the study (2259 men and 1788 women). The Pittsburgh Sleep Quality Index and the duration of insomnia symptoms were used to identify DSM-IV 1–6 month and 6-month insomnia disorders.

**Results:**

The prevalence of DSM-IV defined insomnia disorder was 5.8 %; two-thirds of these case lasted for ≥6 months. The shared correlates for both 1–6 and 6-month insomnia disorders were gender (women), depression and moderate pain. Pulmonary diseases were exclusively associated with 1–6 month insomnia disorder (OR: 2.57, 95 % CI: 1.46–4.52). In contrast, heart disease (OR: 1.73, 95 % CI: 1.21–2.49) and severe pain (OR: 2.34, 95 % CI: 1.14–4.40) were associated with 6-month insomnia disorder.

**Conclusion:**

The prevalence of persistent insomnia disorder is higher than short-term insomnia disorder. Correlates for less persistent and more persistent insomnia disorder appears to be partially different. Duration quantifiers may be important in the identification of the etiology of insomnia and further studies with follow-ups are needed to examine the order of developing insomnia disorder and associated conditions.

## Background

As the architecture of sleep changes along with aging, sleep becomes vulnerable in older adults [1]. Sleep disturbance is a common public health issue in the elderly. Although the prevalence of insomnia symptoms is high in older adults [2–4], insomnia has been more closely associated with comorbidities rather than with age per se [2]. Consequences of poor sleep in older adults include poor health, poor physical function, frequent falls, cognitive impairment and even mortality [5]. Furthermore, it has been found that a bi-directional association exists between heart disease, hypertension, chronic pain, breathing, urinary, gastrointestinal disorders and persistent insomnia [6, 7]. Because persistent insomnia greatly impairs general health and quality of life [8, 9], it is important to examine clinical conditions which correlate with persistence of insomnia in older adults.

During the natural course of insomnia, sleep condition fluctuates over time. Some insomnia episodes are resolved after acute events; some insomnia persists even when the precipitating factors have been resolved. In the general population, only 31.6 to 51.5 % of insomnia remits [10, 11], while 44.9 to 66.1 % of insomnia persists [11, 12] and the recurrence rate reaches as high as 11.8 % [11]. Because the course of insomnia fluctuates and persistence between follow-up points is questionable, it is difficult to evaluate the chronicity of insomnia in longitudinal studies. With respect to older adults, a 3-year, large-scale follow-up study reported a remission rate of insomnia of approximately 50 % in community-dwelling older adults [13]. Nevertheless, there is less knowledge about the natural course of insomnia in older adults. Furthermore, to the best of knowledge, few studies have used a formal diagnostic system to examine the persistence of insomnia in older adults [11, 12]. To prevent insomnia from becoming chronic, it is important to investigate the factors associated with the persistence of insomnia disorder in older adults [14].

In addition, with regard to the definition of persistent insomnia, the duration quantifier is not consistent across various diagnostic systems. According to the Diagnostic and Statistical Manual of Mental Disorders 4th edition (DSM-IV) diagnostic criteria for primary insomnia, insomnia lasting for longer than 1 month is called persistent insomnia [15]. According to the International Classification of Sleep Disorders 2nd edition (ICSD-2) diagnostic criteria for psychophysiological insomnia, insomnia lasting less than 1 month is termed acute insomnia, whereas insomnia lasting for 1 to 6 months is termed sub-acute insomnia. Insomnia lasting for more than 6 months is classified as chronic insomnia [16]. According to the International Classification of Diseases 10th edition (ICD-10) diagnostic criteria, insomnia lasting for longer than 1 month is termed chronic insomnia [17]. The DSM-V diagnostic criteria for insomnia disorder define insomnia of longer than 3 months as persistent insomnia [18]. Insomnia disorder can be classified as acute or chronic according to different duration quantifiers, which imply differential etiologies, associated factors, treatment strategies, and even outcomes. However, the validity of the duration quantifiers of insomnia disorder in older adults has not been examined.

In summary, factors associated with persistence of insomnia disorder in older adults warrant further investigation. The present study aims to identify the prevalence of insomnia disorder in older adults according to different duration quantifiers. The second aim was to examine and compare factors associated with insomnia disorder specified by different duration quantifiers.

## Methods

### Study site and participants

This cross-sectional study was part of the Shih-Pai Sleep Study. The procedure of participant selection has been detailed elsewhere [19, 20]. In short, the participants were older adults (aged ≥65) living in the Shih-Pai area of Taipei City, Taiwan, from 1999 to 2002. In the government household registration system, there were 249,231 people, 65 year-old and older, living in Taipei City in 1999. According to the same system, 9141 older people (aged ≥65) lived in this area. In this population, 1990 people lived in institutions, died before they could be interviewed, moved elsewhere, or could not be contacted. Thus, there were 7151 eligible participants. Data were collected by well-trained study assistants via door-to-door, face-to-face interviews after informed consent. A total of 3104 older adults were unable to be contacted after three attempts or refused to participate; therefore, 4047 eligible adults completed the interview. The response rate was 56.6 %. This study was approved by the institutional review board of the Taipei Veterans General Hospital.

### Demographic data, health behaviors, and medical conditions

All participants completed a questionnaire that assessed the socio-demographic characteristics (age, gender, marital status, educational status, and living status), health behaviors (including smoking and drinking alcohol), and medical diseases (hypertension, diabetes mellitus, heart disease, stroke, gouty arthritis, and pulmonary diseases). In the health behavior, current drinkers were those who had the habit to drink at least once a week. Current smoking was defined as people who still had the habit of smoking during the period of study. Self-report of pharmacologically-treated chronic diseases was confirmed by the receipt of relevant medical treatment of the disease according to their statement during the interview. Information about major chronic diseases was collected, including diabetes mellitus, hypertension, heart diseases (such as coronary artery disease, rheumatic heart disease, etc.), stroke, gouty arthritis and pulmonary diseases (such as asthma, chronic bronchitis, chronic obstructive pulmonary disease, etc.).

The Geriatric Depression Scale-Short Form (GDS-SF) was used to screen for depressive symptoms. The psychometric of Chinese-version of GDS-SF has been shown to have good validity and reliability [21]. The suggested cut-off point for the Chinese-version GDS-SF is 5 and more [22]. Older adults who scored ≥5 on the GDS-SF were classified as having significant depression. Participants who reported sleepiness at work or meals during the daytime were classified as having excessive daytime sleepiness. Pain evaluation is derived from an item on Short Form −36 [23, 24], which aims to assess the severity of pain in the past 1 month. The original response of pain severity is a Likert scale, which consists of: (1) no bodily pain, (2) very mild bodily pain, (3) mild bodily pain, (4) moderate bodily pain, (5) severe bodily pain, (6) very severe bodily pain. Because the data is sparse, we combined the response of “very mild” and “mild” to “mild”, and “severe” and “very severe” to “severe”. Finally, severities of pain were classified into 4 scales: (1) no pain, (2) mild pain, (3) moderate pain, and (4) severe pain. The number of falls in the past year was also recorded. A “single fall” indicated having had only one fall in the past year. If the participants experienced more than one falling accident, the response was coded as “repeated falls”.

### The diagnosis and classification of insomnia disorder

To identify insomnia disorder, the Chinese version of Pittsburgh Sleep Quality Index [25, 26] and DSM-IV criteria for insomnia disorder were used. Chinese version of PSQI has been shown to have good validity in community sample with insomnia disorder [26]. This approach has been adapted to predict the survival of older adults with DSM-IV insomnia disorder in another study [20]. The Pittsburgh Sleep Quality Index is composed of two clusters of questions. The first cluster assesses nighttime symptoms and the second cluster assesses sleep quality and daytime dysfunction.

Cluster 1: Nighttime symptoms

1. Difficultly falling asleep: defined as unable to fall asleep after resting in bed for more than 30 min, or
2. Difficultly maintaining asleep: defined as waking up more than three times during nighttime sleep, or early morning awakening: waking up 2 h earlier than usual.

Any symptoms should occur at least three times per week to qualify for Cluster 1.

Cluster 2: Sleep quality and/or daytime dysfunction

1. Subjectively poor sleep quality (selecting a rating of 3 or 4 on the severity scales) or
2. People who suffered a moderate or greater degree of daytime dysfunction in emotion, working efficacy, and daily living (selecting a rating of 3 or 4 on the severity scales).

If participants met both clusters of symptoms and the duration was for more than 1 month, they met the diagnostic criteria for DSM-IV insomnia disorder. Among the participants qualified as having insomnia disorder, if their insomnia persisted for ≥6 months, they were classified as having a “6-month insomnia disorder.” Participants who met the diagnosis of insomnia disorder with the duration of less than 6 months were classified as having “1–6 month insomnia disorder.”

### Statistical analysis

All statistical analyses were conducted using IBM SPSS Statistics 20 software. Univariate analyses were performed using the chi-square test. Multinomial logistic regressions with forward stepwise method were used to examine the correlates of the 1–6 and 6-month insomnia disorders. Statistical significance was set as *p* < 0.05.

## Results

Table 1 summarizes the basic characteristics of participants and the prevalence of insomnia disorders. Of the participants, 37.6 % were ≥75 years and 55.8 % were male. There was no significant difference between the study subjects and registered data on people older than 65 year-old in Taipei with respect to age (*χ*2 = 1.02, df = 1, *P* = 0.31) and gender (*χ*2 = 1.91, df = 1, *P* = 0.17). Overall, the prevalence of DSM-IV insomnia disorder was 5.8 %; 67.2 % of individuals with identified insomnia disorders had the 6-month insomnia disorder. Specifically, out of all participants, the prevalence of 1–6 month insomnia disorder and 6-month insomnia disorder was 1.9 and 3.9 %, respectively. Women (*p* < 0.001) and single individuals (*p* = 0.005) had a higher prevalence of DSM-IV insomnia disorder. Women had a higher prevalence of insomnia disorder for both duration quantifiers (1–6 month: 2.7 % of women vs. 1.3 % of men; and 6 months: 5.0 % of women vs. 3.0 % of men). Similarly, compared with married participants, single older adults had a higher prevalence of insomnia disorder for both duration quantifiers (1–6 month: 3.0 % of single individuals vs. 1.6 % of married individuals; 6 months: 4.6 % of single individuals vs. 3.6 % of married individuals).

**Table 1:** Demographics and health behavior of participants by different durations of DSM-IV insomnia disorder (*n* = 4047)^a^

			Insomnia status						
	Total		No insomnia		1–6 month insomnia disorder		6-month insomnia disorder		*p-value for X* ^*2*^
	*n* = 4047		*n* = 3794		*n* = 78		*n* = 160		
	n	(%)	n	Rate (%)	n	Rate (%)	n	Rate (%)	
Demographic data									
Age (years)									
≥75	1523	37.6	2371	94.4	46	1.8	94	3.7	0.65
<75	2524	62.4	1421	93.7	32	2.1	63	4.2	
Gender									
Male	2259	55.8	2150	95.6	30	1.3	68	3.0	<0.001
Female	1788	44.2	1643	92.3	8	2.7	89	5.0	
Education									
Illiterate	677	16.7	3162	94.2	63	1.9	131	3.9	0.83
Literate	3370	83.3	632	93.9	15	2.2	26	3.9	
Marital status									
Married	3008	74.3	2838	94.8	47	1.6	109	3.6	0.005
Single, divorced and widowed	1039	25.7	956	92.4	31	3.0	48	4.6	
Living status									
With others	3818	94.3	3583	94.3	74	1.9	144	3.8	0.35
Alone	229	5.7	211	92.5	4	1.8	13	5.7	
Health behavior									
Smoking									
Not current smoker	3354	82.9	3139	94	67	2.0	135	4.0	0.44
Current smoker	693	17.1	655	95.2	11	1.6	22	3.2	
Alcohol									
Not current drinker	3675	90.9	3442	94.1	71	1.9	145	4.0	0.78
Current drinker	372	9.2	352	94.9	7	1.9	12	3.2	

^a^Due to missing data, total numbers of participants in some variables are different

**Fig. 1:**
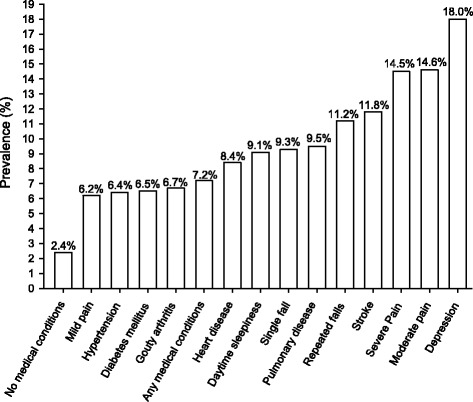
Prevalence of DSM-IV insomnia disorder in various medical conditions. (Chronic diseases were defined as pharmacologically-treated morbidities.)

Table 2 shows the distribution of common medical conditions and their respective prevalence of insomnia disorders. Of the participants, 71.2 % had at least one of the 13 identified medical conditions. Insomnia disorder was significantly associated with heart disease (*p* < 0.001), stroke (*p* = 0.002), pulmonary diseases (*p* = 0.001), depression (*p* < 0.001), excessive daytime sleepiness (*p* = 0.01), pain (*p* < 0.001), and falling (*p* = 0.001). The prevalence of insomnia disorders by common medical conditions ranged from 6.2 to 18.0 %. However, in participants without any medical conditions, the prevalence of a DSM-IV insomnia disorder was only 2.4 %. Figure 1 illustrates the prevalence rates across various medical conditions. Older adults with depression had the highest prevalence of insomnia disorder (18.0 %), followed by those reporting moderate pain (14.6 %), and severe pain (14.5 %).

**Table 2:** Prevalence of DSM-IV insomnia disorder with different durations in various medical conditions (*n* = 4047)^a^

			Insomnia status						
	Total		No insomnia		1–6 month insomnia disorder		6-month insomnia disorder		*p-value for X* ^*2*^
	*n* = 4047		*n* = 3794		*n* = 78		*n* = 160		
	n	(%)	n	Rate (%)	n	Rate (%)	n	Rate (%)	
Medical conditions									
No medical conditions	1161	28.8	1133	97.6	9	0.8	19	1.6	<0.001
Any medical conditions	2868	71.2	2661	92.8	69	2.4	138	4.8	
Chronic diseases^b^									
Diabetes mellitus									
No diabetes mellitus	3523	87.1	3304	94.3	67	1.9	134	3.8	0.78
Diabetes mellitus	524	12.9	490	93.5	11	2.1	23	4.4	
Hypertension									
No hypertension	2467	61.0	2323	94.5	44	1.8	91	3.7	0.50
Hypertension	1580	39.0	1471	93.6	34	2.2	66	4.2	
Heart disease									
No heart disease	3258	80.5	3074	94.8	62	1.9	107	3.3	<0.001
Heart disease	789	19.5	720	91.6	16	2.0	50	6.4	
Stroke									
No stroke	3895	96.2	3660	94.4	74	1.9	143	3.7	0.002
Stroke	152	3.8	134	88.2	4	2.6	14	9.2	
Gouty arthritis									
No gouty arthritis	3735	92.3	3505	94.2	72	1.9	142	3.8	0.67
Gouty arthritis	312	7.7	289	93.2	6	1.9	15	4.8	
Pulmonary diseases									
No pulmonary diseases	3584	88.6	3375	94.6	61	1.7	130	3.6	0.001
Pulmonary diseases	463	9.8	419	90.5	17	3.7	27	5.8	
Depression									
Geriatric depression scale <5	3612	89.3	3437	95.5	60	1.7	102	2.8	<0.001
Geriatric depression scale ≥5	396	9.8	324	82.0	17	4.3	54	13.7	
Clinical conditions									
Excessive daytime sleepiness									
No	3602	89.0	3395	94.6	64	1.8	131	3.6	0.01
Yes	445	11.0	399	90.9	14	3.2	26	5.9	
Pain									
No	2660	66.2	2534	95.3	45	1.7	81	3.0	<0.001
Mild	1070	26.6	1004	93.8	21	2.0	45	4.2	
Moderate	191	4.7	163	85.3	10	5.2	18	9.4	
Severe	103	2.6	88	85.4	2	1.9	13	12.6	
Fall									
No fall	3491	86.3	3294	94.8	59	1.7	121	3.5	0.001
Single fall	398	9.8	361	90.7	13	3.3	24	6.0	
Repeated falls	134	3.3	119	88.8	4	3.0	11	8.2	

^a^Due to missing data, total numbers of participants in some variables are different

^b^Chronic diseases were defined as pharmacologically-treated morbidities

**Fig. 2:**
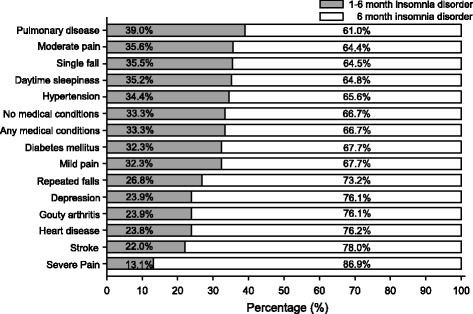
The distribution of DSM-IV insomnia disorder with different duration quantifiers in various medical conditions. (Chronic diseases were defined as pharmacologically-treated morbidities.)

Figure 2 depicts the distribution of insomnia disorder with different duration quantifiers across various medical conditions. The 6-month insomnia disorder was most common among older adults with severe pain (86.9 %) followed by those with stroke (78.0 %), heart disease (76.2 %), depression (76.1 %), gouty arthritis (76.1 %) and repeated falls (73.2 %). In comparison, the 1–6 month insomnia disorder was most common among older adults who had pulmonary diseases (39.0 %), followed by those with moderate pain (35.6 %), single fall (35.5 %) and excessive daytime sleepiness (35.2 %). In this study, the validity of a chronic disease was confirmed by both self-report diagnosis and concurrent medication use (pharmacologically-treated). Because some patients with chronic diseases may receive non-pharmacological intervention, our approach for the confirmation of diagnosis may bias the relationship between chronic diseases and insomnia disorders with different durations. Therefore, the distribution of treatment patterns among different durations of insomnia disorders were further examined with chi-squared tests. The results revealed no significant differences with respect to all chronic diseases.

Table 3 shows the results of multivariate analyses for factors associated with 1–6 and 6-month insomnia disorder. The multinomial stepwise logistic regression showed that 1–6 month insomnia disorder was associated with being a woman (OR: 2.16, 95 % CI:1.33–3.51), having pulmonary diseases (OR: 2.57, 95 % CI: 1.46–4.52), having depression (OR: 2.81, 95 % CI: 1.59–4.96) and moderate pain (OR:2.56, 95 % CI:1.23–5.32). In contrast, 6-month insomnia disorder was associated with being a woman (OR: 1.50, 95 % CI: 1.07–2.10) and having heart disease (OR: 1.73, 95 % CI: 1.21–2.49), depression (OR: 4.68, 95 % CI: 3.24–6.75), or moderate and severe pain (OR: 2.23, 95 % CI: 1.26–3.93 and OR: 2.34, 95 % CI:1.14–4.40) respectively.

**Table 3:** Multinomial logistic regression analysis with forward stepwise method for factors associated with different duration of insomnia disorders

	1–6 month insomnia disorder	6-month insomnia disorder
	OR (95 % CI)	OR (95 % CI)
Gender		
Male	1	1
Female	2.16 (1.33–3.51)	1.50 (1.07–2.10)
Heart disease		
No heart disease	1	1
Heart disease	0.92 (0.51–1.65)	1.73 (1.21–2.49)
Pulmonary disease		
No pulmonary disease	1	1
Pulmonary disease	2.57 (1.46–4.52)	1.54 (0.98–2.41)
Depression		
Geriatric depression scale <5	1	1
Geriatric depression scale ≥5	2.81 (1.59–4.96)	4.68 (3.24–6.75)
Pain		
No	1	1
Mild	1.01 (0.59–1.73)	1.08 (0.74–1.59)
Moderate	2.56 (1.23–5.32)	2.23 (1.26–3.93)
Severe	0.39 (0.05–2.93)	2.34 (1.14–4.40)

Covariates entered the model were age, gender, educational status, marital status, living status, smoking, alcohol use, diabetes mellitus, hypertension, heart disease, stroke, gouty arthritis, pulmonary diseases, depression, excessive daytime sleepiness, different severities of pain, falls

## Discussion

The present study reported the prevalence of DSM-IV defined insomnia disorder and its correlates for different duration quantifiers. The prevalence of DSM-IV insomnia disorder was 5.8 % among home-dwelling older adults, and the insomnia lasted for more than 6 months for two-thirds of this group. However, in healthy participants, the prevalence was only 2.4 %. Although a higher proportion of DSM-IV insomnia disorder tended to last for ≥6 months, factors associated with the 1–6 and 6-month insomnia disorders differed. Gender (women), depression and moderate bodily pain were shared correlates for both 1–6 and 6-month insomnia disorders. Pulmonary diseases were exclusively related to 1–6 month insomnia disorder. In contrast, heart disease and severe pain were exclusively associated with 6-month insomnia disorder. These findings support the importance of examining the validity of duration quantifiers for insomnia disorders. To the best of our knowledge, this is the first study to explore the correlates of insomnia disorder with different chronicities in older adults.

The prevalence of insomnia in older adults varies in the definition of insomnia. A systemic review noted that the prevalence of insomnia symptoms in older adults was 6.9–48.0 %, insomnia symptoms plus daytime symptoms was 9–15 %, poor sleep quality and quantity was 8–18 %, and DSM-IV insomnia criteria was only 4.4–6.4 % [27]. In contrast, in older adults in the Chinese population, the prevalence of insomnia symptoms was about 14.0 to 39.2 % [4, 28, 29] and that of poor sleep quality ranged from 16.0 to 49.7 % [30–35]. In the present study, the prevalence of DSM-IV insomnia disorder was 5.8 %. As far as we know, the prevalence of DSM-IV insomnia disorder has never been surveyed in the Chinese population, except the Shih-Pai cohort [19, 20]. In general, the prevalence of insomnia of various definitions in older Chinese adults was comparable with the worldwide prevalence, including DSM-IV insomnia disorder [27, 36, 37]. Although the effect of the year or cohort may influence the findings of epidemiological studies, the prevalence of insomnia appears to vary more greatly with the definition of insomnia rather than the effect of the year or the cohort [27]. However, large-scale epidemiologic studies for a more recent Chinese cohort of older adults are still necessary to update the information about prevalence and correlates of insomnia disorders with different chronicity.

Insomnia has been observed to have a chronic course and insomnia disorder is more likely to persist than are mere insomnia symptoms. A 1-year follow-up study in Sweden showed that the persistence rate of insomnia was 44.4 % in the general population [11, 12]. Another follow-up study in the United States found persistence rates for insomnia symptoms and diagnosed insomnia in the general population were 37.2 and 66.6 %, respectively [11]. In community-dwelling older adults, only 50 % experienced remission of insomnia symptoms over the 3-year follow-up period [13]. In the present study, because of the cross-sectional design, we could not evaluate the persistence rate of insomnia disorder. However, the high proportion of insomnia disorder with a duration of ≥6 months is a reflection of the persistent nature of insomnia disorder. Further longitudinal studies are necessary to determine the pattern of the course, and associated factors of the chronicity of insomnia disorder in older adults.

Although the cohort for the present study was established during 1999 to 2002, correlates identified for insomnia disorder are similar to those otherwise reported in recent studies [29, 30, 34, 38]. In the regression analyses, gender, physical diseases, depression, and pain stood out as significant correlates of DSM-IV insomnia disorders. In the literature, women, depression, use of hypnotics, respiratory symptoms, poor self-rated health, and somatic symptoms have been reported to correlate with insomnia symptoms in older adults [3, 28]. In contrast, only a few studies have focused on examining the associated factors of insomnia disorder. Furthermore, no studies have compared the factors associated with duration of insomnia disorders.

Being female has been widely identified as a risk indicator across the life span for insomnia, including among older adults. However, it has been argued that the association between being female and insomnia symptoms in older adults is confounded by depression, chronic diseases, living alone, marital status, occupational status, and social support deficits [39]. Nevertheless, in the present study, even after adjusting for various covariates, being female remained a significant correlate for both 1–6 and 6-month insomnia disorders. Therefore, in older adults, being female should be seen as consistent risk indicator for insomnia, at least for DSM-IV insomnia disorder.

In the literature, the association between insomnia and depression has been widely examined [3, 28, 40–43] and a bidirectional relationship between insomnia and depression is also well-known [44]. In parallel, insomnia also predicts the development and recurrence of depression in older adults [42]. Furthermore, persistent sleep disturbance, instead of intermittent sleep disturbance, predicted the recurrence of depression in older adults [41]. On the contrary, although there is no specific research reporting the residual insomnia symptoms in older adults, it has been reported that high proportion of depressive patients have severe insomnia, and insomnia symptoms tend to persist even after the remission of depression [45]. In the present study, although depression was associated with both the 1–6 and 6-month insomnia disorders, the magnitude of the association was greater for the 6-month insomnia disorder. Because of the cross-sectional design of the present study, it is impossible to infer the temporal relationship between DSM-IV insomnia disorder and depression. However, the relationship between depression and insomnia disorder seemed to amplify over time. This finding implied that chronic and severe insomnia in older adults particularly signals the risk of co-occurrence of depression.

Insomnia and pain are also mutually influenced. Pain increases the risk of incident and persistent insomnia [46–48], and sleep disturbance can perpetuate pain [46, 49, 50]. Severe pain in patients with diabetic peripheral neuropathy has been shown to correlate with sleep problems [51]. Furthermore, the impact of sleep on pain accumulates over time; therefore, increased severe pain may be more closely related to persistent insomnia [49]. In the present study, moderate pain was associated with both 1–6 month insomnia disorder and 6-month insomnia disorder; whereas, severe pain was exclusively associated with 6-month insomnia disorder. This finding supported the previous observation that pain influences persistent insomnia and the impact of sleep on pain accumulated over time. Several clinical conditions, including osteoporosis, rheumatoid arthritis, arthrosis, Bechterew’s disease, migraine, and musculoskeletal disorders, were found to cause pain and subsequently result in insomnia in the older adults [52]. In addition, pain sites may also determine the pattern of sleep disturbance. For example, back pain may result in non-refreshing sleep, morning headache may indicate an underlying sleep-disordered breathing, and migraine may hint sleep deprivation [53]. However, there is no study examining the association between underlying causes and specific sites of pain with the persistence of insomnia disorder. In the future, studies that aim to disentangle the relationship between causes and sites of pain and persistence of insomnia disorder are necessary. At least, according to a previous report and our finding, it suggests that pain in older adults with DSM-IV insomnia disorders warrants in-depth survey of the underlying medical causes and specific sites of pain.

Chronic diseases correlate closely with various sleep disturbances in older adults [7, 43]. In the present study, the prevalence of insomnia disorder in healthy participants was not only lower than in older adults with medical conditions, but was also lower than the prevalence reported in the general population [27]. Because both aging and chronic diseases compromise older adults’ ability to sleep, it is imperative to elucidate the link between sleep disturbance and medical comorbidities. In 2003, the “Sleep in America Survey” found that different sleep disturbances might correlate with different categories of self-reported physical conditions. In the “Sleep in America Survey”, depression and heart disease were associated with more general insomnia symptoms. In contrast, pulmonary diseases, obesity, diabetes mellitus, arthritis, stroke, and osteoporosis were associated with other sleep disturbances, such as sleep breathing disorder, snoring, and excessive daytime sleepiness [43]. This finding implied that different chronic diseases might interact with and affect sleep through different pathways. In fact, the clinical course of many physical diseases is chronic in nature. However, according to the features of individual diseases and their treatment modalities, they may influence sleep in either an acute or a chronic pattern.

In the present study, pulmonary diseases were found to be associated with the less persistent, 1–6 month insomnia disorder. On the other hand, heart disease was found to be associated with the more persistent, 6-month insomnia disorder. There are some potential explanations for the differential pattern of comorbidities of 1–6 month and 6-month insomnia disorders. Firstly, many pulmonary diseases influence sleep, such as chronic obstructive pulmonary disease, asthma, and obstructive sleep apnea [54, 55]. Pulmonary diseases and sleep have a bidirectional relationship [56]. When nocturnal symptoms of pulmonary diseases are aggravated, the patients’ sleep quality worsens [57, 58]. In the other direction, sleep disturbance predicted the acute exacerbation of pulmonary diseases and the need for pertinent emergency services [59]. The episodic attacks that characterize many pulmonary diseases may contribute to the link with episodic short-term sleep disturbance. Symptoms of pulmonary diseases such as asthma or chronic obstructive pulmonary disease would aggravate during acute infection or exacerbation [60]. In the multiple logistic regression, pulmonary diseases were only associated with the 1–6 month insomnia disorder, but not with the 6-month insomnia disorder. This finding echoes the above argument about the link between episodic attacks and short-term sleep disturbance.

In contrast, heart disease was exclusively related to 6-month insomnia disorder in the present study. The evidence that insomnia contributes to heart disease is robust [61–64]. Insomnia has been found to increases the risk of incidents of coronary heart disease and elevated cardiovascular mortality. As a result, sleep disturbances are regarded as one of the top ten potentially modifiable risk factors for cardiovascular disease [65]. Insomnia may affect cardiovascular system by having a negative impact on metabolism, endocrine, autonomic nervous system, hemodynamic, cardiac, endothelial, and platelet aggregation functions [66, 67]. Furthermore, insomnia may share some common risk factors with heart disease [62]. This finding may suggest that chronic insomnia seems to be more likely comorbid with diseases in the cardiovascular system.

Although the pattern of correlates for insomnia disorder with different duration of chronicity appears to differ with regard to amount and essence, the interpretation about the differential pattern requires caution. Firstly, the case number of 1–6 month insomnia disorder (*n* = 78) and 6-month insomnia disorder (*n* = 160) were different. The case number of 1–6 month insomnia disorder was around half that of 6-month insomnia disorder. Therefore, the differential pattern between the insomnia disorders with different durations may just reflect the disparity in statistical power. Some potential correlates for 1–6 month insomnia may be underpowered as they failed to turn out statistically significant. Secondly, some patients classified as 1–6 month insomnia disorder might develop 6-month insomnia disorder later. This misclassification was caused by the limitation of diagnostic criteria. This systemic bias will introduce misclassification bias and should be non-differential in essence. Therefore, it would result in a bias toward the null. According to these methodological considerations for the present studies, correlates that specifically appeared as correlates for 1–6 month insomnia disorder, such as pulmonary diseases, are rather robust in evidence, because of both under-power and the bias which is toward the null. In contrast, correlates identified specifically for 6-months insomnia disorder may be erroneous accordingly, such as heart disease and severe pain. Thirdly, our findings merely suggest correlates for older adults with 1–6 month and 6-month insomnia disorder, respectively. Our findings did not suggest predictors to develop 6-month insomnia disorder among individuals with DSM-IV insomnia disorder.

## Limitations

There were several limitations in this study. First, study subjects were home-dwelling older adults who lived in an urban area. Older adults who reside in rural areas and those who have been institutionalized were not included. A previous study found that associated factors differed among older adults living in rural and urban areas [29]. Thus, the study results could only be generalized to home-dwelling older adults living in an urban area. Second, this study was a cross-sectional investigation, which prevents any inference of causal relations between the 1–6 month and 6-month insomnia disorders and their associated factors. Third, according to the study aims and the consideration of statistical power of the present study, only common correlates for insomnia disorder were included into the analyses. However, there remain some important medical diseases, such as genitourinary disease, and cancer, which may correlate with specific durations of insomnia disorders and should be considered in the analyses. It is not possible to reveal whether differential correlation exists between uncollected data on these medical diseases and specific durations of insomnia disorders. Therefore, we could not generalize the results to older adults with these uncollected medical conditions. Furthermore, if these medical diseases correlate with insomnia disorder and distribute unequally in the current recruited variables, the present findings may be further confounded. Therefore, a large-scale prospective study with detailed survey of chronic diseases is necessary to provide a more comprehensive understanding of the relationship between chronic diseases and insomnia disorder. Fourth, in the older adults, exercise can improve sleep quality [68]. On the contrary, physical disability is associated with poor sleep quality [69]. Unfortunately, items related to exercise were not included in our questionnaire. Therefore, we could not analyze the influence of physical activity on insomnia. Fifthly, many commonly used medications, which also impact sleep were not included in the analyses. Although, some confounding effects of these medications may be under-controlled, simultaneous inclusion of chronic diseases and their respective medications may result in over-control. Besides, discarding chronic diseases and only including their medications into the statistical models may also lead to the confounding by disease indication [70]. Therefore, an ingenious statistical or methodological approach to disentangle the complex relationship among chronic diseases, medications, and insomnia disorders should be considered in the future work. Finally, the accuracy of the self-reported duration of insomnia warrants discussion. Although retrospective recall is an inherent feature of sleep studies, the decline of cognitive function in older adults may further bias the subjective reporting of the duration of insomnia. In the present study, cognitive function test for each participant was not available. However, eligible participants, who were able to complete the entire process of data collection for the present study, satisfied cognitive criteria for a near 40-min interview. Therefore, the bias brought by cognitive decline during normal aging might be minimal. In addition, individuals with insomnia have been shown to underestimate their sleep efficacy [71–74]. In the future, it is of interest to examine how cognitive decline and subjective bias of older insomniacs influence the reporting of insomnia duration.

## Conclusion

In home-dwelling older adults, the prevalence of persistent insomnia disorder was higher than short-term insomnia disorder. Correlates for less persistent and more persistent insomnia disorder appeared to be different. Our findings suggested that duration quantifiers may be important in the identification of the etiology of insomnia. Because the complex relationship between insomnia disorder and its correlates, clear clinical recommendations are possible only after the temporal relationship between the development of insomnia disorder and the associated conditions are examined by further follow-up studies in the future.

## Abbreviations

DSM-IV, diagnostic and statistical manual of mental disorders 4th edition; ICSD-2, international classification of sleep disorders 2nd edition; ICD-10, international classification of diseases 10th edition; GDS-SF, the geriatric depression scale-short form; SF-36, short form 36 health survey questionnaire.
